# Analysis of serum calprotectin levels in ménière’s patients and investigation of its effect on disease severity

**DOI:** 10.1371/journal.pone.0340121

**Published:** 2026-01-29

**Authors:** Hüseyin Işık, Deniz Baklaci, Tuba Candar, Duygu Erdem, Ergin Bilgin

**Affiliations:** 1 Department of Otolaryngology, Zonguldak Bülent Ecevit University Hospital, Zonguldak, Türkiye; 2 Department of Biochemistry, Ufuk University Medical Faculty, Ankara, Turkey; University of Connecticut, UNITED STATES OF AMERICA

## Abstract

**Objective:**

This prospective observational study aimed to evaluate serum calprotectin levels in patients with definite Ménière’s Disease (MD) and to investigate their association with symptom duration and disease severity.

**Materials and methods:**

Thirty-nine patients (16 males, 23 females; mean age 37.2 ± 11.9 years) diagnosed with definite MD according to the AAO–HNS criteria and 28 age- and sex-matched healthy controls (14 males, 14 females; mean age 39.2 ± 13.3 years) without vestibular or auditory complaints were included. Individuals with active infection, autoimmune or chronic inflammatory disease, recent antibiotic or steroid use, or other otologic pathologies were excluded. Serum calprotectin levels were measured during an acute vertigo attack in the MD group and once in controls. Between-group differences and correlations with clinical variables were analyzed using appropriate parametric and non-parametric tests.

**Results:**

Mean serum calprotectin levels during acute attack were significantly higher in the MD group than in controls (1031 ± 364 vs. 611 ± 231 ng/mL, *p* < 0.001). Within the MD group, calprotectin levels showed a strong positive correlation with symptom duration (Spearman’s ρ = 0.914, *p* < 0.001). In patients with MD, calprotectin levels decreased significantly after treatment, from 1031 ± 364 to 582 ± 339 ng/mL (mean difference 449 ng/mL, 95% CI 353–545; *t*(38) = 9.46, *p* < 0.001; Cohen’s *d* = 1.51), indicating a large effec*t* size.

**Conclusion:**

Patients with Ménière’s Disease exhibit elevated serum calprotectin levels during acute attacks, which correlate strongly with symptom duration and decrease significantly after treatment. These findings suggest that calprotectin may be a potential inflammatory biomarker associated with disease burden and treatment response in MD; however, longitudinal studies with larger cohorts are needed to determine its diagnostic and prognostic utility.

## Introduction

Ménière’s Disease is a multifactorial, chronic, episodic condition characterized by vertigo, ear fullness, and hearing loss. The peak age of onset is in the 40s, and it is less common in children and patients over 65 years of age. The underlying pathologic condition has been described, but the pathophysiology of the Disease is not yet fully understood. Many factors, including genetic, infectious, autoimmune, and hormonal, are thought to play a role in the aetiology of the Disease. Infectious causes and inflammation play an important role among etiologic factors. Frejo et al. observed that basal levels of proinflammatory cytokines TNF-α, IL-1β, and IL-6 may be increased in some Ménière’s patients. This observation was made after stimulation with lipopolysaccharide (LPS), which served as a positive control for inflammation in patients with Ménière’s Disease and healthy individuals [1]. Several authors have reported a significant improvement in vestibular symptoms with either generic or specific dietary changes in patients with Ménière’s Disease. A possible link between food allergies and Ménière’s Disease symptoms was first suggested in 1923; since then, since the 70s, targeted elimination diets have been recommended for documented food allergies, and evidence has been confirmed that avoidance may prevent an immune-mediated reaction in the inner ear. Calprotectin is a calcium-binding protein predominantly found in neutrophil granulocytes and, to a lesser extent, in monocytes and activated macrophages. It plays a significant role in the innate immune response due to its antimicrobial properties and is widely recognized as a biomarker of inflammation, particularly in inflammatory bowel diseases. Recent studies have suggested that systemic inflammation may contribute to the pathogenesis of Ménière’s Disease, yet the specific role of calprotectin in this context remains unexplored. In studies, fecal calprotectin levels were found to be high in inflammatory bowel diseases, and a decrease in a short time was observed to be a good prognostic indicator of the Disease. Ometto et al., in their study on calprotectin levels in rheumatologic diseases, found that calprotectin serum levels were particularly high in AOSD (adult-onset Still’s Disease). They reported that calprotectin levels in other fluids, such as saliva and synovial fluid, may help diagnose Sjögren’s Syndrome and Gout or Rheumatoid Arthritis, respectively [2]. The role of calprotectin in the inflammatory process has been demonstrated in many studies; however, there are no previous studies on its role in the pathogenesis, diagnosis, and monitoring of Ménière’s Disease.

This study aims to investigate whether serum calprotectin levels are elevated in patients with Ménière’s Disease compared to healthy controls, and to assess whether these levels correlate with disease severity and symptom duration.

## Materials and methods

This study was conducted in accordance with the Declaration of Helsinki, and approval was obtained from the ethics committee. Ethics committee approval for this study was obtained from Zonguldak Bülent Ecevit University, Non-Interventional Clinical Research Ethics Committee (Date: 27/12/2023, Meeting no: 2023/25). All participants provided written informed consent prior to their inclusion in the study.

For the study, 39 patients who applied to Zonguldak Bülent Ecevit University Faculty of Medicine Hospital Ear Nose and Throat Outpatient Clinic with complaints of dizziness, pressure sensation in the ear, and hearing loss and who were diagnosed with ménière’s Disease as a result of audiometry and caloric test, were included in the study as “patient group. The control group was composed of 28 healthy volunteers who postsented to our ENT outpatient clinic with non-inflammatory conditions, such as simple nasal obstruction or benign paroxysmal positional vertigo, and had no known systemic inflammatory, autoimmune, malignant, or active infectious diseases. The standard medical treatment (**high-dose intravenous methylprednisolone,** administered at a dose of **250 mg once daily for 3 days acetazolamide,** at a dose of **250 mg orally, twice daily**) administered to all patients during their acute vertigo attack. Serum calprotectin was studied in the control group of patients who did not have any ear complaints. For the patient group, blood samples were collected during an acute vertigo attack (within 24 hours of symptom onset) and again two weeks after completion of standard medical treatment. For the control group, a single blood sample was obtained during routine outpatient visits.

Serum calprotectin levels were measured using a commercially available ELISA kit (Brand, Model, Manufacturer, Country) in the hospital’s central laboratory. Serum calprotectin levels of the patient and control groups were compared, and the level of calprotectin in Ménière’s Disease and its effect on the severity of the disease were analyzed.

### Statistical analysis

The normality of data distribution was assessed using the Shapiro–Wilk test. Differences in serum calprotectin levels before and after treatment within the Ménière’s Disease (MD) group were analyzed using the paired samples *t*-test. Comparisons of serum calprotectin levels between the MD and control groups were performed using the Mann–Whitney U test because of non-normal distribution. Categorical variables were compared using the chi-square test. Correlations between calprotectin levels and symptom duration were evaluated using Spearman’s rho. A *p*-value < 0.05 was considered statistically significant.

### Findings

The study group consisted of 39 patients with Ménière’s Disease (16 males, 23 females), and the control group consisted of 28 healthy individuals (14 males, 14 females). The mean age was 37.2 ± 11.9 years in the study group and 39.2 ± 13.3 years in the control group, with no statistically significant difference between the groups (*p* = 0.625) (Table 1). In the Ménière’s Disease group, serum calprotectin levels measured during an acute attack were significantly higher than post-treatment levels. Calprotectin decreased from 1031 ± 364 ng/mL before treatment to 582 ± 339 ng/mL after treatment (mean difference 449 ng/mL, 95% CI 353–545; *t*(38) = 9.46, *p* < 0.001; Cohen’s *d* = 1.51), indicating a large effect size (Table 2). When calprotectin levels during acute attack in the Ménière’s Disease group were compared with those of the control group, patients with Ménière’s Disease exhibited significantly higher levels (1031 ± 364 vs. 611 ± 231 ng/mL, *p* < 0.001) (S1 Fig, Table 3). Within the Ménière’s Disease group, a strong positive correlation was observed between pre-treatment serum calprotectin levels and symptom duration (Spearman’s ρ = 0.914, *p* < 0.001) (Table 4).

**Table 1:** Demographic characteristics of participants.

Characteristic	Total (n = 67)	Control (n = 28)	Ménière’s Disease (n = 39)
Sex, n (%) – Males	30 (44.8%)	14 (20.9%)	16 (23.9%)
Sex, n (%) – Females	37 (55.2%)	14 (20.9%)	23 (34.3%)
Age (years), mean ± SD	38.0 ± 12.5	39.2 ± 13.3	37.2 ± 11.9
p value (age)			0.625

**Table 2:** Serum calprotectin levels before and after treatment in Ménière’s Disease group.

Measure	N	Mean (ng/mL)	Median (ng/mL)	SD	Mean difference (ng/mL)	95% CI (lower–upper)	p value/ Cohen’s d
Pre-treatment calprotectin	39	1031	931	364	–	–	–
Post-treatment calprotectin	39	582	506	339	449	353–545	< 0.001/ 1.51

**Table 3:** Comparison of serum calprotectin levels between Ménière’s Disease and control groups.

Group	N	Mean (ng/mL)	Median (ng/mL)	SD	p value*
Ménière’s Disease (acute attack)	39	1031	931	364	< 0.001
Control	28	611	652	231	

* p value based on Mann–Whitney U test.

**Table 4:** Correlation between serum calprotectin levels and symptom duration in Ménière’s Disease.

Variables	Spearman’s rho	p value
Pre-treatment calprotectin vs. symptom duration	0.914	< 0.001

## Discussion

Ménière’s Disease (MD), or idiopathic endolymphatic hydrops, is an inner ear disorder characterized by episodes of spontaneous vertigo, fluctuating hearing loss, aural fullness, and tinnitus. Although its prevalence increases with age and it is most commonly seen between the fourth and seventh decades, its etiology remains unclear. Anatomical factors, allergy, trauma, infections, autoimmune mechanisms, and genetic predisposition have all been implicated. Several studies support a role for immune and inflammatory mechanisms in MD. For example, Keleş et al. reported significantly higher total and specific IgE levels in patients with MD compared with healthy controls, suggesting a contribution of allergic sensitization to disease pathophysiology [3]. In addition, a large retrospective study by Hoa et al. demonstrated that hearing fluctuations are common in MD and are associated with future hearing fluctuations and hearing loss, highlighting the dynamic and heterogeneous nature of the disease course [4].

Biomarkers reflecting inner ear pathology have also been investigated. Avallone et al. found that serum levels of OTOLIN-1, an inner ear–specific structural protein, were significantly higher in patients with MD and sudden sensorineural hearing loss than in healthy controls, whereas no difference was observed in vestibular neuritis [5]. This suggests that acute inner ear disorders may be accompanied by detectable changes in serum biomarkers, although OTOLIN-1 itself is not a marker of systemic inflammation.

Calprotectin is a neutrophil-derived protein that is markedly elevated in infectious and inflammatory conditions, including inflammatory bowel disease, and is widely used as a marker of increased intestinal permeability and mucosal inflammation. Beyond the gastrointestinal tract, calprotectin has been implicated in various chronic inflammatory diseases. In a literature review, Manfredi et al. emphasized that calprotectin (cCLP) may serve as a biomarker reflecting both local and systemic inflammation and could have prognostic implications in chronic rheumatic diseases [6]. These observations support the broader concept that calprotectin is a sensitive indicator of inflammatory activity across different organ systems.

In our study, we observed that serum calprotectin levels measured during acute attacks were significantly higher in patients with MD than in healthy controls. The mean serum calprotectin level in the MD group (1031 ± 364 ng/mL) was clearly above that of the control group (611 ± 231 ng/mL), and this difference was statistically significant (*p* < 0.001). The mean value in the control group is consistent with previously reported levels for healthy individuals, which are typically below 1 µg/mL (1000 ng/mL), in line with the findings of Carnazzo et al. [7]. Furthermore, we found a strong positive correlation between calprotectin levels during acute attacks and symptom duration (Spearman’s ρ = 0.914, *p* < 0.001), suggesting that patients with a longer history of symptoms tend to exhibit higher inflammatory activity as reflected by serum calprotectin.

Our results are in keeping with previous work linking calprotectin to inner ear disorders. Kuzucu et al. reported significantly higher serum calprotectin levels in patients with idiopathic sudden sensorineural hearing loss (ISSHL) compared with healthy controls and found that higher calprotectin levels were associated with more severe hearing loss and poorer response to treatment [8]. While ISSHL and MD are distinct clinical entities, both conditions may involve inflammatory mechanisms affecting the inner ear. The parallels between our findings and those of Kuzucu et al. support the hypothesis that calprotectin may reflect the inflammatory component of inner ear pathology rather than being disease-specific.

We also demonstrated a significant reduction in serum calprotectin levels after treatment in the MD group. Calprotectin decreased from 1031 ± 364 to 582 ± 339 ng/mL, with a large effect size (Cohen’s *d* = 1.51). This finding indicates that calprotectin levels are responsive to clinical improvement and may be linked to changes in disease activity. Di Berardino et al. previously showed that patients with symptomatic MD had increased intestinal permeability and elevated fecal calprotectin compared with asymptomatic patients and healthy controls [9]. Taken together with our results, these data suggest that MD may have a systemic inflammatory or gut–inner ear axis component in at least a subset of patients, rather than representing a purely localized inner ear disorder.

Our study has several limitations. First, the relatively small sample size and single-center design may limit the generalizability of our findings. Second, although we found significant differences in mean serum calprotectin levels between patients and controls, our study was not designed to establish a diagnostic threshold. Given that calprotectin is a non-specific marker of inflammation, the mean value observed in our patient group (1031 ng/mL) cannot be used as a standalone diagnostic cut-off for MD. Third, we measured calprotectin only once in controls and only during acute attacks in patients; repeated measurements during interictal periods were not performed. Longitudinal studies that compare calprotectin levels in the same patients during both ictal and interictal phases would provide a better understanding of temporal changes and their relationship to symptom fluctuations. Fourth, our assessment of disease severity relied primarily on symptom duration. A more comprehensive evaluation incorporating attack frequency, audiometric parameters, and patient-reported outcomes or functional handicap scores would allow a more robust assessment of the relationship between calprotectin and disease severity. Finally, baseline characteristics such as BMI, smoking status, alcohol consumption, and comorbidities, which may influence systemic inflammatory markers, were not systematically recorded and could not be adjusted for in our analyses.

In conclusion, our findings demonstrate that serum calprotectin levels are significantly elevated in patients with Ménière’s Disease during acute attacks compared with healthy controls and decrease following standard treatment. The strong positive correlation between calprotectin levels and symptom duration suggests an association with cumulative disease burden and inflammatory activity rather than providing direct evidence of prognosis. Serum calprotectin may therefore represent a potential inflammatory biomarker in MD, particularly in the context of research studies investigating disease mechanisms and treatment responses. However, large-scale, multicenter prospective studies with longitudinal follow-up are required to validate these findings, to determine whether calprotectin can contribute to risk stratification or monitoring in clinical practice, and to explore its role alongside other clinical and audiological indicators in the long-term management of Ménière’s Disease.

## Supporting information

S1 Fig Serum calprotectin levels before and after treatment in ménière’s disease. (JPG)
