# Supplementary figures and images for: Analysis of serum calprotectin levels in ménière’s patients and investigation of its effect on disease severity
Source: PLoS One. 2026 Jan 29;21(1):e0340121. doi: 10.1371/journal.pone.0340121 (PMC12854440; doi:10.1371/journal.pone.0340121)

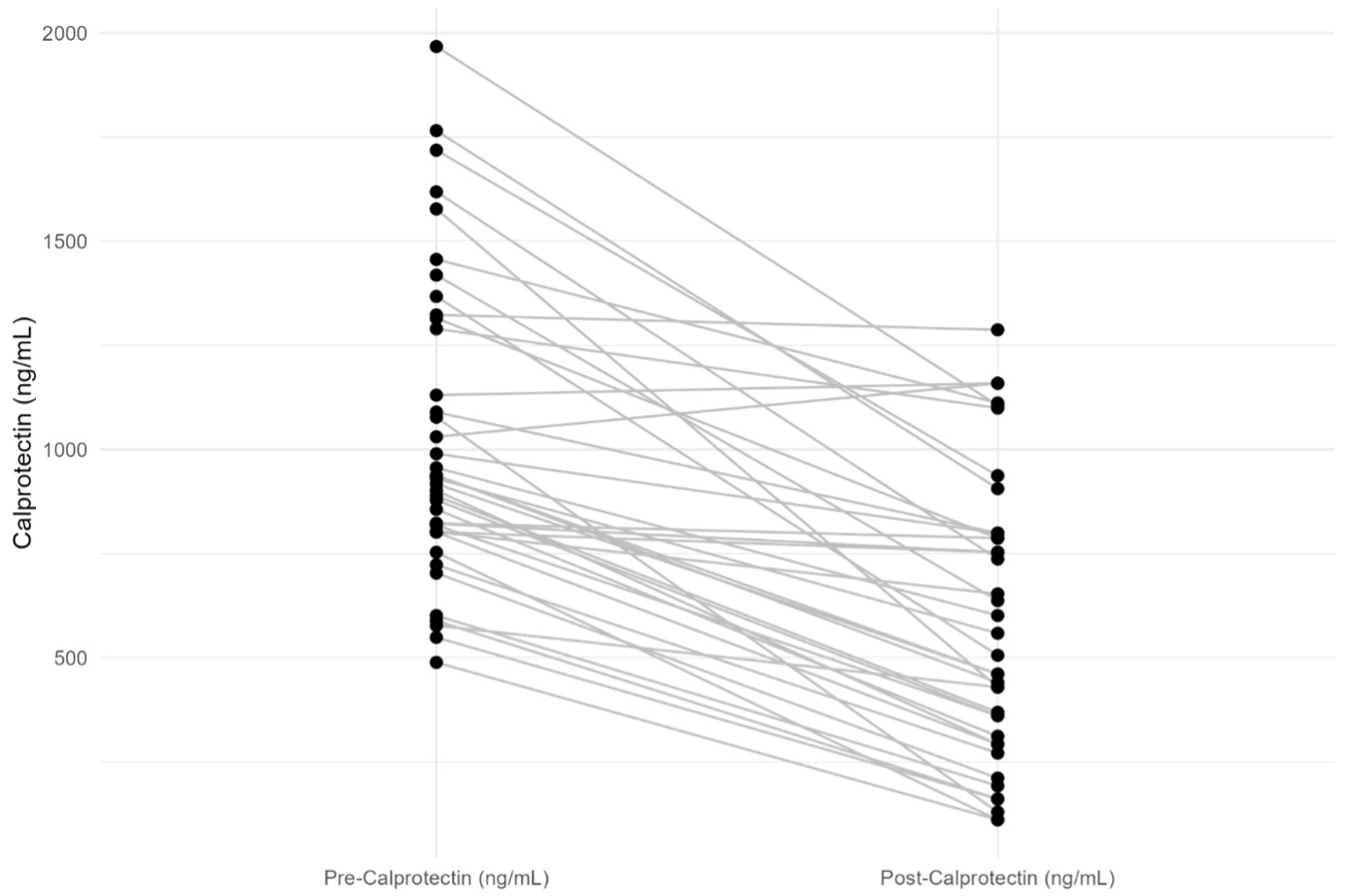

Supplement: S1 Fig — (JPG) [file pone.0340121.s001.jpg]
